# Precise Detection of Cataracts with Specific High‐Risk Factors by Layered Binary Co‐Ionizers Assisted Aqueous Humor Metabolic Analysis

**DOI:** 10.1002/advs.202105905

**Published:** 2022-05-26

**Authors:** Chenjie Yang, Aizhu Miao, Chaochao Yang, Chuwen Huang, Haolin Chen, Yongxiang Jiang, Chunhui Deng, Nianrong Sun

**Affiliations:** ^1^ Department of Chemistry Institue of Metabolism and Integrate Biology (IMIB) Zhongshan Hospital Fudan University Shanghai 200433 China; ^2^ Eye Institute and Department of Ophthalmology, Eye & ENT Hospital Fudan University Shanghai 200031 China; ^3^ Department of Gastroenterology and Hepatology Zhongshan Hospital Fudan University Shanghai 200032 China

**Keywords:** aqueous humor, cataracts, diabetes, high myopia, laser desorption/ionization mass spectrometry, metabolic fingerprints, nanomaterials

## Abstract

Diabetes and high myopia as well‐known high‐risk factors can aggravate cataracts, yet clinical coping strategy remains a bottleneck. Metabolic analysis tends to be powerful for precisely detection and mechanism exploration since most of diseases including cataracts are accompanied by metabolic disorder. Herein, a layered binary co‐ionizers assisted aqueous humor metabolic analysis tool is proposed for potentially etiological typing and detection of cataracts, including age‐related cataracts (ARC), cataracts with diabetes mellitus (CDM), and cataracts with high myopia (CHM). Startlingly, taking advantage of the optimal machine learning algorithm and all metabolic fingerprints, 100% of accuracy, precision, and recall rates are achieved for arbitrary comparison between groups. Moreover, 11, 9, and 7 key metabolites with explicit identities are confirmed as markers of discriminating CDM from ARC, CHM from ARC, and CDM from CHM, and the corresponding area under the curve values of validation cohorts are 0.985, 1.000, and 1.000. Finally, the critical impact of diabetes/high myopia on cataracts is revealed by excavating the change levels and metabolic pathways of key metabolites. This work updates the insights of prevention and treatment about cataracts at metabolic level and throws out huge surprises and progresses metabolic diagnosis toward a reality.

## Graphical abstract

In this work, the well‐designed layered binary co‐ionizers‐assisted laser desorption/ionization mass spectrometry acquires high‐quality metabolic fingerprints from aqueous humor samples with different cataract patients including age‐related cataracts, cataracts with diabetes mellitus, and cataracts with high myopia. With the optimal machine learning algorithm and key features, 100% of accuracy, precision, and recall rates are achieved in pairwise discrimination.

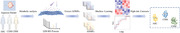

## 1 Introduction

Cataracts are a class of blinding eye diseases with many symptoms such as lens protein denaturation^[^ ^1^ ^]^ and metabolic disorders,^[^ ^2^ ^]^ which are triggered by various causes including age,^[^ ^3^ ^]^ high myopia,^[^ ^4^ ^]^ diabetes,^[^ ^5^ ^]^ intoxication and radiation, and so on. In the past two decades, the new cases of cataracts are continusouly more than 10 million worldwide, ranking the first cause of blindness all the time.^[^ ^6^ ^]^ Currently, the effective treatment toward visually prominent cataracts is intraocular lens implantation. However, the relatively mature phacoemulsification technology can only improve the vision of pure age‐related cataracts (ARC) to a certain extent,^[^ ^7^ ^]^ and is often unsatisfactory for most of those cataracts accompanied by other high‐risk factors such as diabetes and high myopia, since these cataract patients have normally developed severe fundus changes and retinopathy when clinically diagnosing at first time owing to their earlier onset and faster progress.^[^ ^5^, ^8^ ^]^ Moreover, current clinical examination for cataracts with high‐risk factors needs professional physicians to combine slit lamp microscopy, fundus examination, patient's history, and so on, which is cumbersome and time‐consuming.^[^ ^9^ ^]^ In theory, molecular analysis can perfectly realize the precise diagnosis regardless of personal clinical experience, which is still in the exploratory stage. As a consequence, an advanced tool with high specificity and rapidness is urgently needed for those cataracts accompanied by other potential causes, with ability to timely monitor cataracts with high‐risk factors at the molecular level for preventing acute deterioration or improving prognosis.

Different from deoxyribonucleic acids and proteins that are limited by the epigenetic events and posttranslational modifications, respectively, metabolites as the end products of the organism metabolism are related to phenotype directly.^[^ ^10^ ^]^ Exploration on cataracts at the metabolic level has been considered as a beneficial way to understand its occurrence and development.^[^ ^11^ ^]^ However, traditional metabolomics researches focus on crystalline lens, restricted by the difficult sample accessibility.^[^ ^12^ ^]^ Aqueous humor (AH), a transparent liquid surrounding the anterior and posterior chambers of the eyes, plays an important role in maintaining intraocular pressure, supplying nutrients and antioxidants to the lens, as well as removing metabolic wastes from the lens.^[^ ^13^ ^]^ Tentative researches have revealed that metabolite abnormalities present in AH of cataracts with diabetes or high myopia, suggesting that AH may contain useful information with enough strong ability to reflect eye‐related lesion.^[^ ^14^ ^]^ Moreover, AH is more easily accessible compared to the crystalline lens. Therefore, AH metabolic analysis‐based tool is highly anticipated in the respect of detecting and monitoring cataracts with different high‐risk factors.

Mass spectrometry (MS) has been widely used in metabolomics researches, and stands out for high sensitivity and high resolution.^[^ ^15^ ^]^ As an outstanding representative, laser desorption/ionization MS (LDI‐MS) features by high throughput (hundreds of samples at the same time), amazing speed (within seconds), and simple sample preparation (even without pretreatment process), showing great potential in extracting metabolic patterns.^[^ ^16^ ^]^ In particular, the requirement of sample volume at the nanoliter level lays a sturdy foundation for the analysis of precious samples like AH. Notably, the performance of LDI‐MS is decided by the matrix to a large extent. Various nanomaterials have been designed as matrices for LDI‐MS, such as carbon materials,^[^ ^17^ ^]^ metal‐organic frameworks,^[^ ^18^ ^]^ metals,^[^ ^19^ ^]^ and metal oxides,^[^ ^20^ ^]^ which possess obviously remarkable background elimination ability compared to conventional organic matrices. Among them, bulk titania has been regarded as a considerable one due to strong UV absorption, chemical stability, nontoxicity, and low price.^[^ ^21^ ^]^ In general, bulk titania adsorbs laser to generate electron–hole pairs on its surface, then analytes are protonated due to hole trapping.^[^ ^22^ ^]^ However, on one hand, the recombination rate of electrons and holes is faster than the trapping rate of charge carriers and interfacial charge transfer rate on the surface of titania. On the other hand, the exterior instead of the interior of titania is more preferable to capture electrons.^[^ ^23^ ^]^ Hence, the ionization efficiency of bulk titania is usually discounted and fails to meet demand of practical application.

For solving the above problem, hybrid matrix appears continuously, especially the introduction of noble metal nanoparticles can well enhance ionization efficiency. Noble metal nanoparticles can not only serve as electron sinks to suppress the surface recombination rate of electrons and holes, but also provide stronger laser adsorption because of surface plasmon resonance effect. While bulk carriers can merely offer the limited space for loading noble metal nanoparticles, easily resulting in only a slight enhancement in ionization efficiency, or even the appearance of metal aggregation producing background interference. In this regard, we proposed a layered binary co‐ionizer consisting of hierarchically porous titania nanosheets and Au nanoparticles for LDI‐MS analysis. Compared to bulk titania, hierarchically porous titania nanosheets will expose more exterior surface to decrease the recombination rate of charge carriers on the surface of binary co‐ionizers, provide more sites for carrying more Au nanoparticles and enough space for uniform arrangement of Au nanoparticles, thereby significantly improving the ionization efficiency and avoiding the aggregation of Au nanoparticles. Moreover, the layered binary co‐ionizers will provide more available reaction sites for trapping analytes for high‐efficiency ionization. Given the above, the layered binary co‐ionizers‐assisted LDI‐MS and machine learning were applied to reveal metabolic differences and screen out potential metabolic markers through depicting AH metabolic fingerprints (AHMFs) of ARC, cataracts with diabetes mellitus (CDM), and cataracts with high myopia (CHM). This work will inspire more devotion in the field of developing co‐ionizer‐based metabolic analysis tools toward precisely personalized diagnosis and treatment.

## 2 Results and Discussion

### 2.1 Construction of Metabolic Extraction Method on Layered Binary Co‐Ionizers

**Figure 1:**
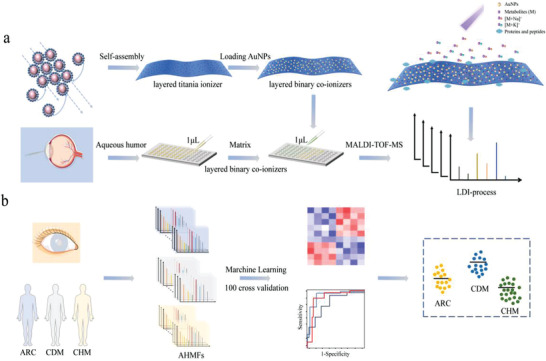
The workflow of precise detection of cataracts with different high‐risk factors. a) The synthesis and application of layered binary co‐ionizers in extracting AHMFs. b) Machine learning for deep exploration of cataracts with different high‐risk factors based on metabolic features.

**Figure 2:**
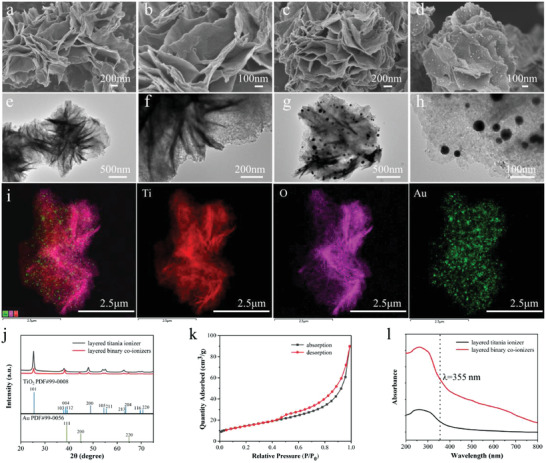
Characterization of layered titania ionizer and layered binary co‐ionizers. The FESEM images of a,b) layered titania ionizer and c,d) layered binary co‐ionizers. The FETEM images of e,f) layered titania ionizer and g,h) layered binary co‐ionizers. i) Overlapped figure and corresponding element mapping images. j) X‐Ray Powder diffraction patterns of layered titania ionizer and layered binary co‐ionizers. k) Nitrogen adsorption−desorption isotherms of layered binary co‐ionizers. l) The UV–vis spectra of layered titania ionizer and layered binary co‐ionizers.

The emphasis of metabolic extraction method is the preparation of excellent layered binary co‐ionizers. The preparation of layered binary co‐ionizers mainly includes two steps (**Figure** **1**a). The first step is to fabricate layered mesoporous titania nanosheets through a successive bottom‐up assembly process at the accurately controllable monomicelle level.^[^ ^24^ ^]^ During this process, the treatment of self‐assembly precursor solution is quite significant. Briefly, the initial precursor solution consisting of tetrahydrofuran and titanium source tetrabutyl titanate and amphiphilic triblock copolymer Pluronic F127 was turned into the viscous gel via evaporation at 45 °C, the gel was then dispersed into the mixture solution of ethanol and glycerol for further treatment at 100 °C. The second step is to apply the resultant layered mesoporous titania nanosheets as scaffolds to sustain the growth of Au nanoparticles. For realizing the uniform arrangement of Au nanoparticles on the surface, the nanosheets were dispersed into chloroauric acid solution under magnetic stirring, followed by sodium citrate. This can effectively avoid the aggregation of Au nanoparticles and the detailed process is described in Text S1, Supporting Information. The field emission scanning electron microscope (FESEM) and field emission transmission electron microscope (FETEM) were first employed to characterize the morphology of layered co‐ionizers and dispersion of Au nanoparticles. As seen in **Figure** **2**a,b, the conspicuous hierarchical stretching structure of titania nanosheets can be observed in the FESEM images, and from the corresponding FETEM images in Figure 2e,f, the average width of the nanosheets is around 1 µm. These are favorable for the adhesion of gold nanoparticles compared with spherical titania or single‐layer titania. Moreover, Figure 2c,d shows the FESEM images of layered binary co‐ionizers. As expected, the titania ionizer maintains hierarchical structure after modification of Au nanoparticles, and the Au nanoparticles with the average particle size of 15 nm evenly scatter on the layered titania nanosheets (Figure 2g,h). The corresponding element mapping images in Figure 2i well demonstrate the uniform distribution of Ti, O, and Au elements in layered binary co‐ionizers. Additionally, the X‐Ray powder diffraction and X‐ray photoelectron spectroscopy were applied to verify the synthesis of layered binary co‐ionizers. As shown in Figure 2j, the well‐defined diffraction peaks at 25.5°, 37.8°, 48.0°, 53.8°, 55.2°, and 62.9°could be indexed to the 101, 004, 200, 105, 211, and 204 reflections of anatase, demonstrating the highly crystalline anatase phase (space group I41/amd) of the 2D mesoporous layered titania ionizer. And the well‐defined diffraction peaks at 38.2°, 44.4°, and 64.576° could be indexed to the 111, 200, and 220 reflections of gold, illustrating the well‐design structures of Au nanoparticles.

Furthermore, the Nitrogen adsorption‐desorption isotherms in Figure 2k reveal the characteristic type IV curves with distinguishable capillary condensation step at *P*/*P*_0_ = 0.5 ≈ 1.0, suggesting layered titania ionizer contains uniform mesopores. The Brunauer–Emmett–Teller (BET) surface area and pore volume of the 2D mesoporous layered titania ionizer are estimated to be 105 m^2^ g^−1^ and 0.35 cm^3^g^−1^, respectively. The large surface area provides enough space for adhesion of plentiful Au nanoparticles and their uniform arrangement, and the abundant mesopores lead to the exposure of more exterior surface for reducing the recombination rate of charge carriers. All these will be beneficial to the enhancement of ionization efficiency. Finally, the optical property of layered binary co‐ionizers was tested, which was the most important prerequisite for an excellent matrix. From Figure 2l, layered titania ionizer exhibits the absorption property close to the wavelength at 355 nm that is the common laser wavelength of LDI‐MS. Compared to the single layered titania ionizer, the introduction of Au nanoparticles remarkably enhances the UV absorption, which means the new layered binary co‐ionizers indeed will own superior ionization ability toward analytes.

**Figure 3:**
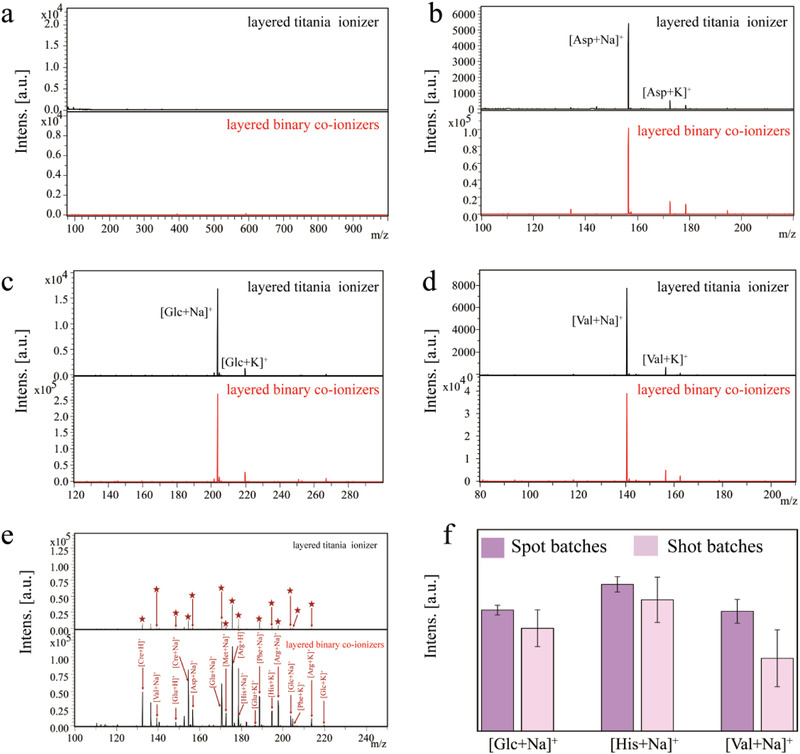
Standard metabolite analysis and batch selection for LDI detection. a) Direct MS spectra of layered titania ionizer and layered binary co‐ionizers. b–d) The analysis of Glc (1 mm), Asp (1 mm), and Val (1 mm) with layered titania ionizer and layered binary co‐ionizers as matrices respectively. e) The MS spectra of mixture of nine metabolites including Asp, Val, Glc, Glu, Phe, Arg, Met, His, and Cre, and the corresponding adducts. f) The intensity of [Glc + Na]^+^, [His + Na]^+^, and [Val + Na]^+^ from different batches of spots or shots (*n* = 5).

Inspired by the above characteristic results, the performance of layered binary co‐ionizers was briefly evaluated by using standard metabolites. At first, interference‐free property in low mass range is an important advantage of nanomaterial matrices in metabolic analysis. In this work, we estimated the background of MS spectra of layered titania ionizer and layered binary co‐ionizers without utilization of metabolites. As shown in **Figure** **3**a, both of MS spectra under 1000 Da are clean. Then, in order to test the analysis performance of layered binary co‐ionizers assisted LDI‐MS toward small molecules, an exploratory MS evaluation was implemented using glucose (Glc), aspartic acid (Asp), and valine (Val) as sample molecules. From Figure 3b–d, it is obviously observed that the signal intensities of Glc, Asp, and Val are enhanced by an order of magnitude when using layered binary co‐ionizers, indicating the good ionization ability of layered binary co‐ionizers toward low‐abundance metabolites. Furthermore, the mixture of nine metabolites including Asp, Val, Glc, glutamic acid (Glu), phenylalanine (Phe), arginine (Arg), methionine (Met), histidine (His), and creatine monohydrate (Cre) was also employed to test the ionization ability of layered titania ionizer and layered binary co‐ionizers. The MS spectra in Figure 3e show all the adducts are detected with enhanced intensities by using layered binary co‐ionizers as matrices. Also, layered binary co‐ionizers acquire more low‐abundance metabolites with stronger signals compared to previous reports.^[^ ^25^ ^]^

At last, we estimated the reproducibility of data acquisition from the batches of spots or batches of shots through recording the average intensities of five MS spectra of [Glc + Na]^+^, [His + Na]^+^, and [Val + Na]^+^. It is worth noting that we adopted a complete scan mode during laser processing in this work to realize an accumulation effect for heightening data reliability. Theoretically, this kind of mode will lead the concentration of analytes to gradually decrease along with each scan, resulting in poor reproducibility of batches of shots. The data acquisition results are displayed in Figure 3f. As expected, the average signal intensities from batches of spots of [Glc + Na]^+^, [His + Na]^+^, and [Val + Na]^+^ fluctuate in controllable range, the relative standard deviation (RSD) values of which are 4.00%, 5.02%, and 9.90% respectively. However, the average signal intensities from batches of shots of [Glc + Na]^+^, [His + Na]^+^, and [Val + Na]^+^ are obviously lower than that from batches of spots, the corresponding RSD values are 17.8%, 17.2%, and 39.0% respectively, affirming the aforehand guess. To sum up, the data acquisition from the batches of spots will be adopted in the AH metabolic analysis.

### 2.2 Extraction of AHMFs for Optimizing Machine Learning Algorithms

**Figure 4:**
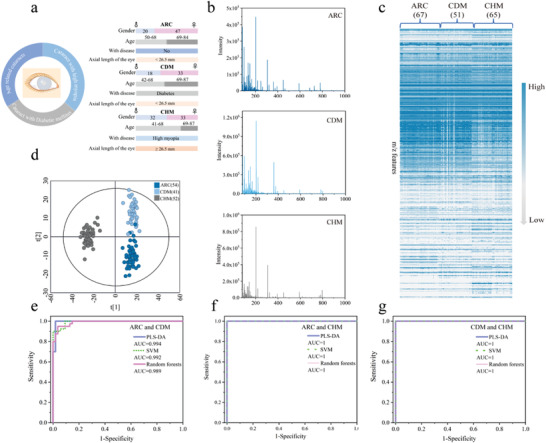
The records of AHMFs and the algorithm optimization. a) The clinical information of 183 cataract patients including 67 ARC, 51 CDM, and 65 CHM. b) Representative metabolic fingerprints of ARC, CDM, and CHM, respectively. c) The total AHMFs of 183 cataract patients. d) The PLS‐DA model of the discovery cohort of ARC, CDM, and CHM. e) The PLS‐DA, SVM, and Random forests models for the discrimination between ARC and CDM. f) The PLS‐DA, SVM, and Random forests models for the discrimination between ARC and CHM. g) The PLS‐DA, SVM, and Random forests models for the discrimination between CDM and CHM.

As mentioned before, the current mature phacoemulsification technology can work for vision improvement of pure ARC to a certain extent, however, it is quite unsatisfactory for most of CDM or CHM that are easily to miss the optimal treatment time, leading to the strong desire for other treatment protocols. Therefore, it is necessary to develop advanced tools to try molecular analysis of cataracts with different high‐risk factors to explore possible treatment protocols, or realize the detection of these cataracts at an early stage to reduce surgical trauma and economic burden. In this work, we attempted to establish the layered binary co‐ionizers assisted LDI‐MS for AHMFs extraction to conduct the precise detection of cataracts with different high‐risk factors (Figure 1b). We collected 183 AH samples from different cataracts with high‐risk factors, including 67 ARC, 51 CDM, and 65 CHM. The detailed information of three groups is displayed in **Figure** **4**a and Table S1, Supporting Information, including gender, age, and the related medical history. Notably, statistical calculation illustrates significant difference of age exists between CHM and ARC/CDM (Table S2, Supporting Information), and the former is younger, indicating that CHM cataracts are probably caused by high myopia, at least partly. All the AHMFs in the *m*/*z* region of 80 to 1000 Da were directly extracted by layered binary co‐ionizers assisted LDI‐MS without any pretreatment. Typical MS spectra of these three groups are shown in Figure 4b, and the total 183 AHMFs are presented in Figure 4c, from which the preliminary feature distribution can be clearly observed. Initially, we randomly selected 80% of samples from each group as a discovery cohort (54 ARC, 41 CDM, and 52 CHM), and the rest as a validation cohort. For tentatively evaluating the discrimination ability of these AHMFs toward cataracts with different high‐risk factors, we chose the partial least‐squares discrimination analysis (PLS‐DA) model as a classifier for the discovery cohort of ARC, CDM, and CHM. As seen in the Figure 4d, the three groups gather in the respective areas within 95% confidence interval (*R* ^2^ *Y*(cum) = 0.991, *Q* ^2^(cum) = 0.863), indicating the great potential of these AHMFs in detection of cataracts with different high‐risk factors. Moreover, based on the 80–20 rule of all the extracted features, 100% of accuracy, precision, and recall rates are achieved for respective discrimination toward ARC versus CDM, CDM versus CHM, and ARC versus CHM.

**Figure 5:**
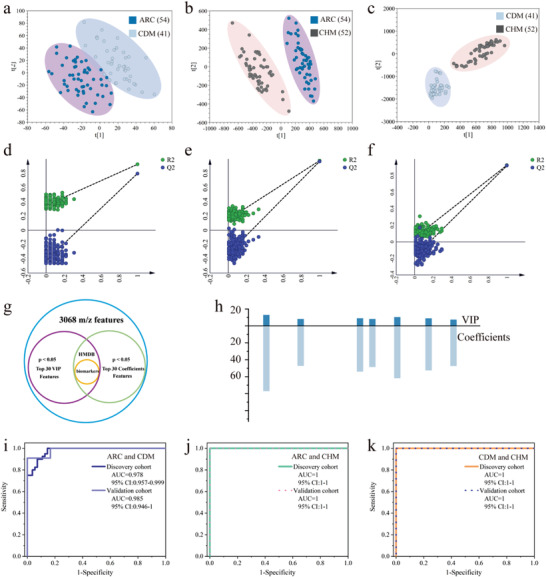
Precise detection of cataracts with different high‐risk factors and the screening and evaluation of key metabolites. a) The PLS‐DA model of ARC and CDM. b) The PLS‐DA model of ARC and CHM. c) The PLS‐DA model of CDM and CHM. d) The 200 permutations of PLS‐DA between ARC and CDM. e) The 200 permutations of PLS‐DA between ARC and CHM. f) The 200 permutations of PLS‐DA between CDM and CHM. g) The selection criteria for biomarkers. h) The VIP scores and coefficients distribution of the nine biomarkers between ARC and CHM. i) ROC curves of discovery cohort and validation cohort of ARC and CDM. j) ROC curves of discovery cohort and validation cohort of ARC and CHM. k) ROC curves of discovery cohort and validation cohort of CDM and CHM.

Encouraged by the above primary results, we further selected various machine learning algorithms including linear support vector machine (SVM) and Random forests and PLS‐DA as classifiers, with aim to pick out the optimal model that can provide better support for the subsequent screening of differential metabolites. In detail, based on AHMFs from discovery cohort, we compared the discrimination performance of PLS‐DA, SVM, and Random forests comprehensively by building receiver operating characteristic (ROC) curves. The ROC curves of ARC versus CDM, ARC versus CHM, CDM versus CHM are presented in Figure 4e–g. Surprisingly, for ARC versus CDM, the area under the curve (AUC) values of PLS‐DA, SVM, and Random forests are 0.994, 0.992, and 0.989, respectively. Among them, PLS‐DA model presents slightly superior discrimination performance. The PLS‐DA model for ARC versus CDM is displayed in **Figure** **5**a (*R* ^2^ *Y*(cum) = 0.924, *Q* ^2^(cum) = 0.795), and the result of 200 permutations is presented in Figure 5d where all green (*R* ^2^) points at left are less than 0.4 and the blue (*Q* ^2^) points at left are lower than *X‐*axis, illustrating PLS‐DA model is reliable. The exciting results suggest significant difference of these AHMFs that can be applied to conduct discrimination and detection of ARC and CDM. More surprisingly, for ARC versus CHM, or CDM versus CHM, the three models present the same strong distinguishing ability, with AUC values up to 1. The PLS‐DA models for ARC versus CHM (*R* ^2^ *Y*(cum) = 0.975, *Q* ^2^(cum) = 0.963), and CDM versus CHM (*R* ^2^ *Y*(cum) = 0.964, *Q* ^2^(cum) = 0.953) are displayed in Figure 5b,c, from which two respective clusters can be observed perfectly. The results of 200 permutations corresponding to ARC versus CHM, or CDM versus CHM in Figure 5e,f confirm the reliability of PLS‐DA model in discrimination of cataracts with different high‐risk factors. The above results fill us with great enthusiasm to further explore AH metabolic difference among various high‐risk cataracts and with full confidence in establishing credible marker panel. In view of the discrimination results of ARC versus CDM, PLS‐DA model is finally selected to carry out deeper excavation of metabolite information.

### 2.3 Establishment of Credible Marker Panels for Precise Detection of Cataracts with Different High‐Risk Factors

To establish the credible marker panels for precisely discriminating cataracts with different high‐risk factors, we attempted to give prominence to significant metabolites in different groups. The variable importance for the projection (VIP) and coefficient score are taken into consideration, both of which are the important measure in PLS‐DA model. In addition to the requirement of the top 30 VIP scores as well as the top 30 coefficient scores, the *p*‐value < 0.05 is also included in the selection criteria as seen in Figure 5g. The features with top 30 VIP and coefficient scores in PLS‐DA models of ARC versus CDM, ARC versus CHM, and CDM versus CHM are respectively presented in Figures S1–S3 and Table S3, Supporting Information. In detail, 27 features, 30 features, and 30 features are respectively screened out for ARC versus CDM, ARC versus CHM, and CDM versus CHM. According to the specific 27 features, the ROC curves of ARC versus CDM in Figure S4, Supporting Information indicate that the AUC value is 0.998 for discovery cohort with 95% confidence interval (CI) of 0.992–1, and 0.993 for validation cohort with 95% CI of 0.971–1. In addition, the accuracy, precision and recall rates are estimated to be 91.7%, 84.6%, and 100%, respectively, when choosing CDM as truly positive specimens. Similarly, according to the specific 30 features, the ROC curves of ARC versus CHM andCDM versus CHM in Figures S5,S6, Supporting Information indicate that the AUC value is 1 for both discovery and validation cohorts, with 95% CI of 1–1. In addition, the accuracy, precision, and recall rates are all estimated to be 100% when choosing CHM as truly positive specimens.

In order to clarify the details of these features, the human metabolome database (HMDB) was applied to match these key metabolites. Finally, eleven key metabolites for ARC versus CDM, nine key metabolites for ARC versus CHM, and seven key metabolites for CDM versus CHM are confirmed. These metabolite features are bolded in Table S3, Supporting Information and the corresponding detail information is presented in Table S4, Supporting Information. The VIP and coefficient scores of the nine specific metabolites for ARC versus CHM, as well as those for ARC versus CDM, CDM versus CHM are presented in Figure 5h and Figure S7, Supporting Information. We further attempted to estimate the significance of these key features with explicit identities in discrimination of different high‐risk cataracts through ROC curves. As displayed in the ROC curves of ARC versus CDM in Figure 5i, the AUC value is 0.978 for discovery cohort with 95% CI of 0.957–0.999, and 0.985 for validation cohort with 95% CI of 0.946–1, presenting slight decrease compared to the result of 27 specific features. Moreover, based on the 80–20 rule of key metabolite features, the accuracy, precision, and recall rates achieve 91.7%, 84.6%, and 100% respectively when choosing CDM as truly positive specimens, which are identical to that based on 27 specific features. Analogously, the ROC curves of ARC versus CHM in Figure 5j show that both AUC values reach 1 for both discovery and validation cohorts with the 95% CI of 1–1. More importantly, 100% of accuracy, precision, and recall rates are attained for respective discrimination toward ARC versus CHM. The ROC curves of CDM versus CHM in Figure 5k show that both AUC values also reach 1 for both discovery and validation cohorts with the 95% CI of 1–1. And the accuracy, precision, and recall rates are estimated as 95.8%, 90%, and 100% for respective discrimination toward CDM versus CHM (CHM as truly positive specimens). These results confirm the remarkable significance of key metabolites in discriminating CDM from ARC, discriminating CHM from ARC, and discriminating CDM from CHM. All the above are quite exciting, highly demonstrating the models are extremely reliable and own excellently precise prediction for different high‐risk cataracts.

**Figure 6:**
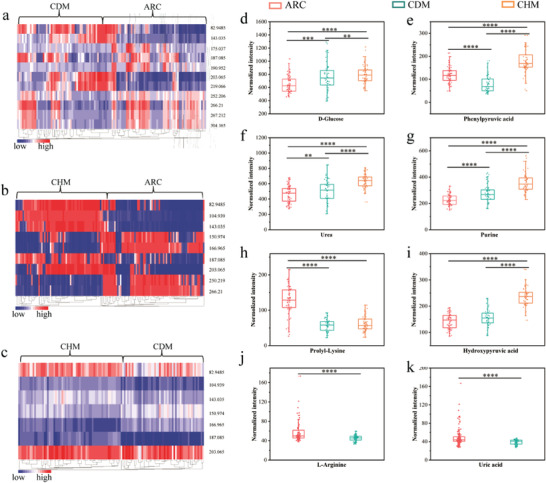
The heatmaps in pairs and the normalized intensity of the matched key metabolites in ARC, CDM, and CHM. a) The heatmap of key metabolites of ARC and CDM. b) The heatmap of key metabolites of ARC and CHM. c) The heatmap of key metabolites of CDM and CHM. d–g) The shared significant metabolites including D‐glucose, phenylpyruvic acid, urea, and purine in ARC, CDM, and CHM. h) The shared proly‐Lysine in ARC and CDM or CHM. i) The shared hydroxypyruvic acid in CDM and ARC or CHM. Two key metabolites in ARC and CDM including j) L‐Arginine and k) uric acid. The significance of the comparisons is presented by ^**^ *p* < 0.05, ^***^ *p* < 0.001, and ^****^ *p* < 0.0001.

Furthermore, the heatmaps in pairs are plotted as presented in **Figure** **6**a–c, the conspicuous differences of key metabolites among different high‐risk cataracts can be observed. We explored the importance of several key metabolites in different high‐risk cataracts comprehensively through their signal intensities (Figure 6d–k), as well, the possible metabolic pathways of key metabolites (Figure S8–S10, Supporting Information). The box plots for the shared key metabolites in ARC, CDM, and CHM, including D‐glucose (*m*/*z* 203.065, [M + Na]^+^), phenylpyruvic acid (*m*/*z* 187.085, [M + Na]^+^), urea (*m*/*z* 82.9485, [M + Na]^+^), and purine (*m*/*z* 143.035, [M + Na]^+^), are presented in Figure 6d–g. According to previous reports, oxidation of lens crystalline proteins and retina is the major contributor to the occurrence of different types of cataracts,^[^ ^26^ ^]^ for which the sustaining high‐glucose concentration takes the main responsibility. The lens and its surroundings are the body parts mostly affected by hyperglycemia that can increase oxidative stress of thus leading to irreversible damage to the retina.^[^ ^27^ ^]^ From Figure 6d, D‐glucose indeed presents up‐regulation in both CDM and CHM compared to ARC. This is also consistent with the fact that diabetes itself is a severe metabolic disorder characterized by hyperglycemia,^[^ ^28^ ^]^ and the uncontrollable glucose metabolism (normally glucose up‐regulation) is known as one of high‐risk factors for myopia.^[^ ^29^ ^]^ As aforementioned, CHM cataracts are probably caused by high myopia, the similar change of D‐glucose between CHM and CDM indicates the diabetes may have become a great threat to cataracts. Additionally, it has been reported that the disorder of phenylalanine is significantly associated with diabetes.^[^ ^28^ ^]^ From Figure S10–S12, Supporting Information, phenylalanine biosynthesis (*p* = 0.02) and phenylalanine metabolism (*p* = 0.05) stick out as the most significant metabolic pathways in pairwise comparison between three groups. Thus, phenylpyruvic acid (*m*/*z* 187.085, [M + Na]^+^) as the direct metabolite of phenylalanine is bound to play a crucial role in the occurrence and progress of CHM, which can be seen from Figure 6e that phenylpyruvic acid presents obvious up‐regulation in CHM compared to ARC and CDM. Noteworthy, phenylpyruvic acid presents obvious down‐regulation in CDM compared to ARC, indicating the certain difference of progress mechanism of CDM from CHM and the exploration significance of precise detection of cataracts with different high‐risk factors. Analogously, the up‐regulation of urea (*m*/*z* 82.9485, [M + Na]^+^) and purine (*m*/*z* 143.035, [M + Na]^+^) in Figure 6f,g appears as anticipated in CDM and CHM compared to ARC, since they represent the occurrence of retinopathy,^[^ ^30^ ^]^ suggesting the significance of early detection of cataracts that are easily exacerbated by other high‐risk factors.

Apart from oxidative stress, non‐enzymatic binding of glucose to proteins (glycation) is regarded as the most important mechanism in the initiation and progression of diabetic cataracts.^[^ ^31^ ^]^ In particular, free amino groups at the side chains of lysine and arginine residues in proteins are the most important candidates for binding.^[^ ^32^ ^]^ Depending on this mechanism, L‐lysine has been an effective medicine against high‐glucose damages and stresses.^[^ ^33^ ^]^ As displayed in Figure 6h, compared to ARC, the intensity of proly‐Lysine (*m*/*z* 266.21, [M + Na]^+^) is significantly down‐regulated in CHM, which is possibly related to the high concentration of glucose. Meanwhile, the approximate down‐regulation of proly‐Lysine in CDM is also observed, indirectly echoing with the consistency of change trend of glucose concentration in CDM and CHM (Figure 6d) and manifesting the menace of diabetes toward cataracts again. In addition, reports have pointed out that 3‐hydroxypyruvic acid (*m*/*z* 104.0110, [M + H]^+^) plays a crucial role in destabilizing hypoxia inducible factor and inducing the irreversible injury of the retina,^[^ ^34^ ^]^ which is another representative one associated with high glucose.^[^ ^35^ ^]^ As displayed in Figure 3, 6, 3‐hydroxypyruvic acid uniformly presents up‐regulation in CDM and CHM compared to ARC, and more up‐regulation in CHM. Besides, we inspected two key metabolites in ARC and CDM as displayed in Figure 6j,k. From Figure S10, Supporting Information, arginine biosynthesis (*p*‐value = 0.002) is the most significant metabolic pathway of key metabolites discriminating CDM from ARC, namely, L‐arginine plays significant roles in the discrimination of these two groups. The mechanism is possibly similar to L‐lysine as proposed above that the free amino groups serve as candidates for binding.^[^ ^32^ ^]^ Thereby, to some extent, the L‐arginine may also be an effective medicine for the treatment of cataracts.^[^ ^31^ ^]^ Specifically, L‐Arginine (*m*/*z* 175.037, [M + H]^+^) in Figure 6j presents down‐regulation in CDM, with different degrees but similar tendency to L‐lysine. As for uric acids (*m*/*z* 190.952, [M + Na]^+^) in Figure 6k, a slight increase can be observed in ARC compared to CDM, which exactly agrees with previous reports.^[^ ^36^ ^]^ Also, uric acid has been reported as an independent risk factor of diabetic retinopathy,^[^ ^37^ ^]^ and as an antioxidant presenting decrease in diabetic patients compared to non‐diabetic ones.^[^ ^38^ ^]^ All these demonstrate the reliability of key metabolites, in other words, the extremely practical operability and the promotion value of our proposed tool.

## 3 Conclusion

In conclusion, we developed a layered binary co‐ionizers assisted AH metabolic analysis tool for realizing precise detection of cataracts with different high‐risk factors. In this work, the layered binary co‐ionizers containing hierarchically porous titania nanosheets well made up for the defects of traditional bulk titania and improved ionization efficiency toward metabolites, through constructing layered porous structure to provide more loading space for the uniformly scattered Au nanoparticles and expose more exterior surface for electron trapping. Benefitting from the combination of hierarchically porous titania nanosheets and Au nanoparticles, AHMFs were successfully extracted from 183 AH samples including 67 ARC, 51 CDM, and 65 CHM. Remarkably, with the application of the 80–20 rule to all samples, the AUC values of PLS‐DA, SVM, and Random forests models based on all AHMFs were 0.994, 0.992, and 0.989 for the discrimination of ARC and CDM, and the AUC values of the three models are up to 1 for the discrimination of ARC and CHM, CDM and CHM, respectively, which was quite high and fully proved the significant value of AH metabolites in detection of cataracts. When applying the PLS‐DA model to respective discrimination between ARC and CDM, CDM and CHM, and ARC and CHM, 100% of accuracy, precision, and recall rates can be achieved based on all AHMFs. Moreover, when applying the important measure (the top 30 VIP scores, top 30 coefficient scores, *p*‐value < 0.05) in PLS‐DA model to rank the significance of metabolic features, 27, 30, and 30 features were screened out for discrimination of ARC and CDM, CDM and CHM, ARC and CHM. Excitingly, with these filtrated features in pairwise discrimination, the accuracy, precision, and recall values were about 91.7%, 84.6%, and 100%, respectively when choosing the CDM (ARC as control) as truly positive specimens, or all were 100% when choosing the CHM (ARC or CDM as control) as truly positive specimens. Besides, the corresponding AUC values of discovery cohorts are 0.993 (ARC and CDM), 1 (ARC and CHM), and 1 (CDM and CHM). Additionally, with the help of HMDB, 11, 9, and 7 key metabolites with explicit identities were confirmed among the above corresponding filtrated features. More excitingly, with these key metabolites in pairwise discrimination, the accuracy, precision, and recall rates achieved 91.7%, 84.6%, and 100% respectively (CDM as truly positive specimens, ARC as control), or all 100% (CHM as truly positive specimens, ARC as control), or 95.8%, 90%, and 100% (CHM as truly positive specimens, CDM as control). Similarly, the corresponding AUC values of discovery cohorts demonstrated excellent prediction capability, 0.985 for ARC versus CDM, 1 for ARC versus CHM, and 1 for CDM versus CHM. The matching results of metabolic pathways further demonstrated the reliability of key metabolites. Furthermore, we explored the possible molecular mechanisms of up and down‐regulation of eight significant metabolites specifically in different high‐risk cataracts at the metabolic level. All the above demonstrated the great values of layered binary co‐ionizers assisted AH metabolic analysis in precise detection of cataracts with different high‐risk factors. In a word, this work can not only provide guidance for the clinical understanding of the molecular function mechanism of different factors toward cataracts but also provide an important reference value for the treatment and prevention of cataracts with high‐risk factors. More importantly, this work gives us full confidence that the precise diagnosis and typing of cataracts with diabetes or high myopia can be a reality.

## 4 Experimental Section

All experimental information has been provided in Experimental Section, Supporting Information, including chemicals and materials, sample collection and preparation, preparation of analyte solutions, LDI‐MS analysis, and statistical analysis. All the 183 AH samples were collected from Eye Institute and Department of Ophthalmology, Eye and ENT Hospital, Fudan University. This study obeyed the ethical standards. Furthermore, it followed the Declaration of Helsinki and was approved by the Ethics Committee of Eye & ENT Hospital of Fudan University (2020126‐1). Besides, all volunteers and patients involved in the research agreed to provide samples, and informed written consent of all participants were obtained.

## Conflict of Interest

The authors declare no conflict of interest.

## Supporting information

Supporting Information

## Data Availability

The data that support the findings of this study are available from the corresponding author upon reasonable request.
