# Supplementary material for: Precise Detection of Cataracts with Specific High‐Risk Factors by Layered Binary Co‐Ionizers Assisted Aqueous Humor Metabolic Analysis
Source: Adv Sci (Weinh). 2022 May 26;9(21):2105905. doi: 10.1002/advs.202105905 (PMC9313487; doi:10.1002/advs.202105905)
Supplement: Supplementary file 1 — Supporting Information [file ADVS-9-2105905-s001.pdf]

ADVANCED SCIENCE
Open Access

Supporting Information

for *Adv. Sci.*, DOI 10.1002/advs.202105905

Precise Detection of Cataracts with Specific High-Risk Factors by Layered Binary Co-Ionizers Assisted Aqueous Humor Metabolic Analysis

Chenjie Yang, Aizhu Miao, Chaochao Yang, Chuwen Huang, Haolin Chen, Yongxiang Jiang, Chunhui Deng* and Nianrong Sun**

Supporting Information

Precise Detection of Cataracts with Specific High-risk Factors by Layered Binary Co-ionizers Assisted Aqueous Humor Metabolic Analysis

Chenjie Yang,[+] Aizhu Miao,[+] Chaochao Yang, Chuwen Huang, Haolin Chen, Yongxiang Jiang, Chunhui Deng*, Nianrong Sun**

C. Yang, C. Yang, H. Chen, Prof. C. Deng
Department of Chemistry, Institue of Metabolism & Integrate Biology (IMIB), Zhongshan Hospital, Fudan University Shanghai 200433, China.
E-mail: chdeng@fudan.edu.cn

A. Miao, Y. Jiang

Eye Institute and Department of Ophthalmology, Eye & ENT Hospital, Fudan University, Shanghai, China; NHC Key Laboratory of Myopia (Fudan University); Key Laboratory of Myopia, Chinese Academy of Medical Sciences, Shanghai 200031, China

E-mail: yongxiang.jiang@fdeent.org

C. Huang, Dr. N. Sun
Department of Gastroenterology and Hepatology, Zhongshan Hospital, Fudan University, Shanghai, 200032, China.
E-mail: sunnianrong@fudan.edu.cn

EXPERIMENTAL SECTION

Text S1

Synthesis of layered binary co-ionizers. The synthesis of layered titania ionizer was carried out according to the reference[1], with slight difference. The ultimately collected white nanoparticles were calcined in a muffle furnace to obtain the layered titania ionizer. Then, 100 mg layered titania ionizer and 1.3 mL of 10 mg mL^{-1} $AuCl_4$ were dispersed into 100 ml deionized water, and the mixed solution was heated to boiling. After that, 7 mL 38.8 mM of trisodium citrate was rapidly added into the flask under magnetic stirring, followed by reflux for fifteen minutes. Finally, after keeping stirring for two hours at room temperature, the products were collected by centrifugation and dried in a vacuum overnight.

Chemicals and Materials. Trisodium citrate dihydrate, tetrahydrofuran, and glycerol were purchased from Sinopharm chemical reagent. Tetrabutyl titanate, Pluronic F127 (PEO106PPO70PEO106, Mw = 12600 g mol^{-1}), D-glutamic acid, L-phenylalanine, L-valine, L-aspartic acid, creatine monohydrate, L-arginine, D-methionine, L-histidine, and D-glucose were purchased from Sigma-Aldrich. 2,5-dihydroxybenzoic acid (DHB) and a-cyano-4-hydroxycinnamic acid (CHCA) were purchased from Adamas.

Samples Collection and Preparation. In this work, all the 183 aqueous humor samples were collected from Eye Institute and Department of Ophthalmology, Eye & ENT Hospital, Fudan University. All the patients were diagnosed as the patients with cataract, and the scales of all three groups of cataract patients were recorded using LOCS III, only those moderate cataracts were taken into this work. Moreover, all these patients included in this study performed fundus examinations and have no surgical contraindications, making sure the low interference of fundus changes and retinopathy as far as possible. Especially, only patients without diabetic retinopathy (NDR) or mild non-proliferative diabetic retinopathy (NPDR) were included, while severe NPDR and proliferative diabetic retinopathy (PDR) were excluded. Additionally, the patients with an eye axis greater than 26.5 mm and without history of diabetes were taken into CHM

group (65); the patients with a clear history of diabetes and without high myopia were taken into CDM group (51); the patients without diabetes and high myopia were taken into ARC group (67). Aqueous humor samples were transported in an insulation box filled with dry ice and placed in -80°C storage immediately. Before LDI-MS analysis, the freezing sample was thawed at 4°C.

Preparation of Analyte Solutions. The layered titania ionizer and layered binary co-ionizers were dispersed in deionized water to obtain uniformly dispersed solutions (1 mg mL^{-1}). L-phenylalanine, L-valine, creatine monohydrate, L-arginine, D-glutamic acid, D-methionine, glucose, and L-histidine solutions were prepared with deionized water respectively and the concentration is controlled to 1×10^{-3} M. The concentration of the metabolite in the mixed solution is also controlled to 1×10^{-3} M.

LDI-MS Analysis. In this wok, only one microliter is taken from all the standard molecule solutions or the aqueous humor samples and the volume of layered titania ionizer and layered binary co-ionizers is also 1 μL. First, 1 μL analytes were pipetted onto a stainless-steel target plate. After being dried at room temperature, 1 μL layered titania ionizer or layered binary co-ionizers were pipetted to cover the plate spot. After being dried at room temperature, the samples were analyzed on a MALDI-TOF MS (UlrafleXtreme MALDI-TOF/TOF MS) in a positive mode and the intensity of laser was 70% and the molecular weight from 80 to 1000 Da was recorded.

Statistical Analysis. The raw data was extracted on the matched Flexanalysis 3.4 software provided by Bruker. The peak extraction, alignment, and average were processed by MALDIquant package on R to build a matrix of m/z signals.[2] Data were shown as the mean ± s.d. with n = 67/51/65 (ARC/CDM/CHM) for clinical indexes. Other univariate statistical analyses in this work were performed using SPSS software version 20.0 (IBM Corp., Armonk, New York), including one-way anova for age comparison, and Chi-square test for sex comparison (Table S1). All significance level was set as 5%. Figures were prepared using Origin software (OriginLab) and SIMCA 14.1 (MKS Umetrics, Umeå, Sweden). The partial least-squares discrimination analysis (PLS-DA), linear support vector machine (SVM), the random forest, and the receiver operating characteristic (ROC) curves were generated at Metaboanalyst 4.0 (McGill

University, Montreal, Canada, https:// www.metaboanalyst.ca/). The random selection of the discovery cohort and the validation cohort were performed on python 3.6.3. The metabolite search was performed at Human Metabolome Database (https://hmdb.ca/).

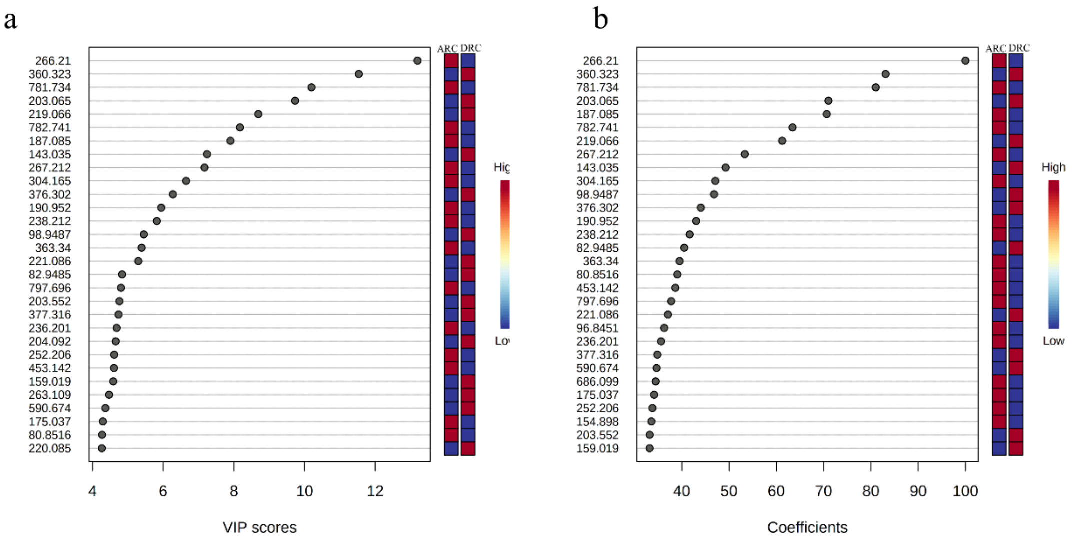

Figure S1. (a) The top 30 VIP scores of the PLS-DA model between ARC and CDM. (b) The top 30 coefficients of the PLS-DA model between ARC and CDM.

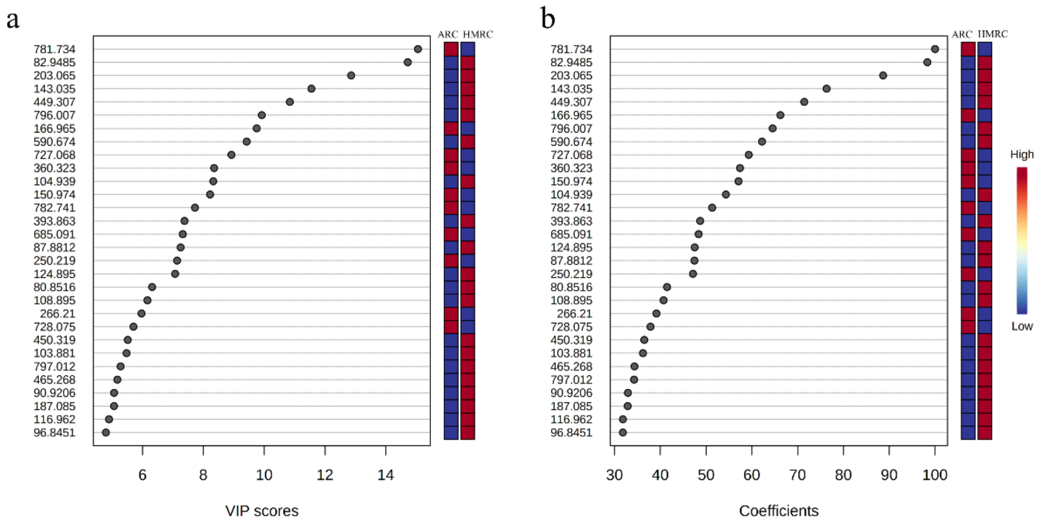

Figure S2. (a) The top 30 VIP score features of the PLS-DA model between ARC and CHM. (b) The top 30 coefficients score features of the PLS-DA model between ARC and CHM.

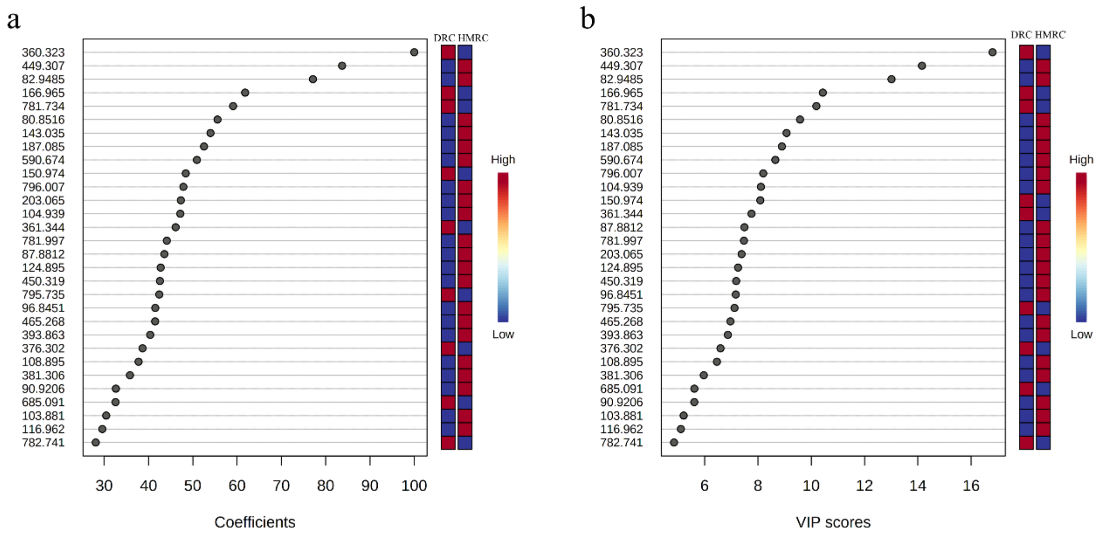

Figure S3. (a) The top 30 VIP score features of the PLS-DA model between CDM and CHM. (b) The top 30 coefficients score features of the PLS-DA model between CDM and CHM.

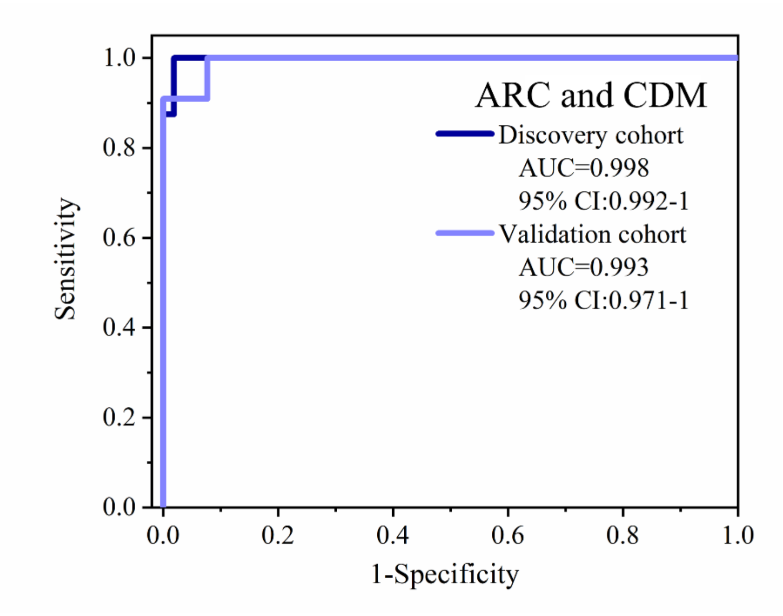

Figure S4. The ROC curves of the ARC and CDM according to 27 specific features.

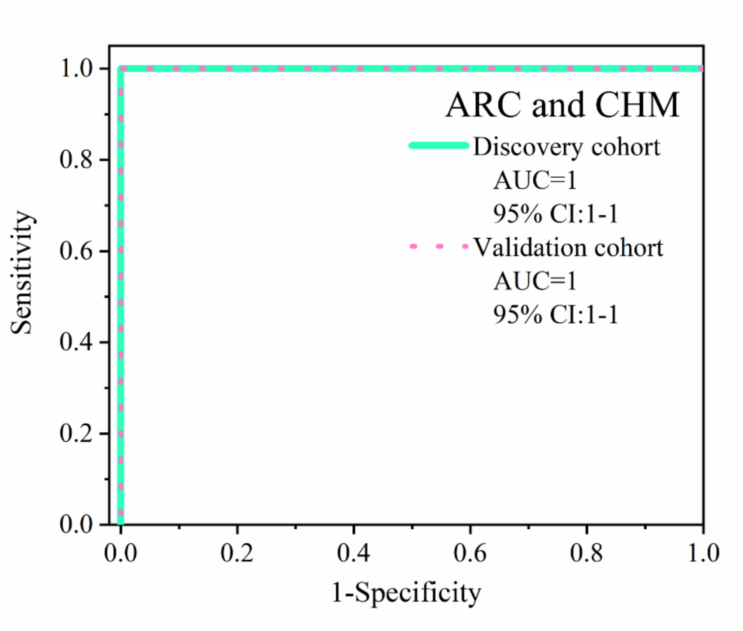

Figure S5. The ROC curves of ARC and CHM according to 30 specific features

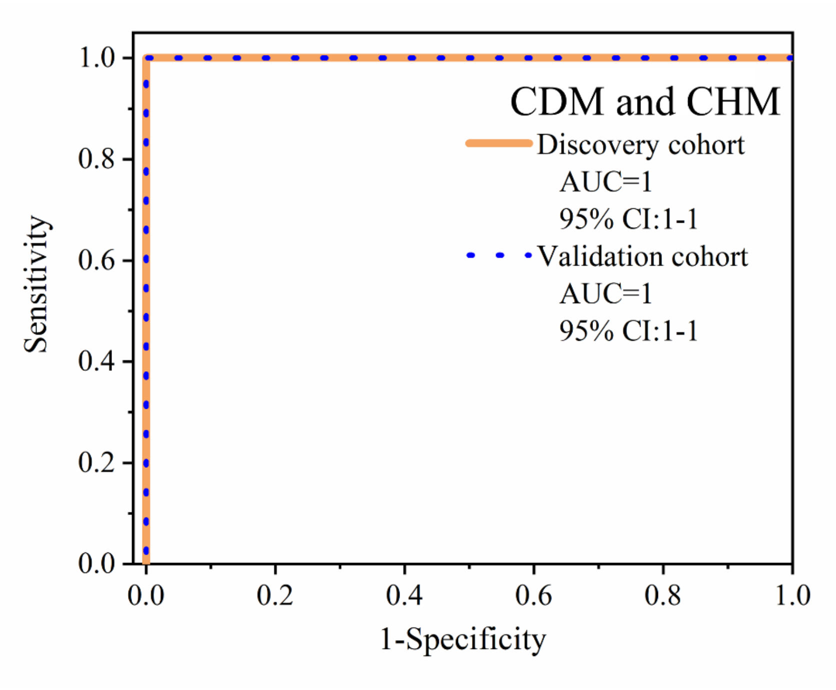

Figure S6. The ROC curves of CDM and CHM according to the 30 specific features.

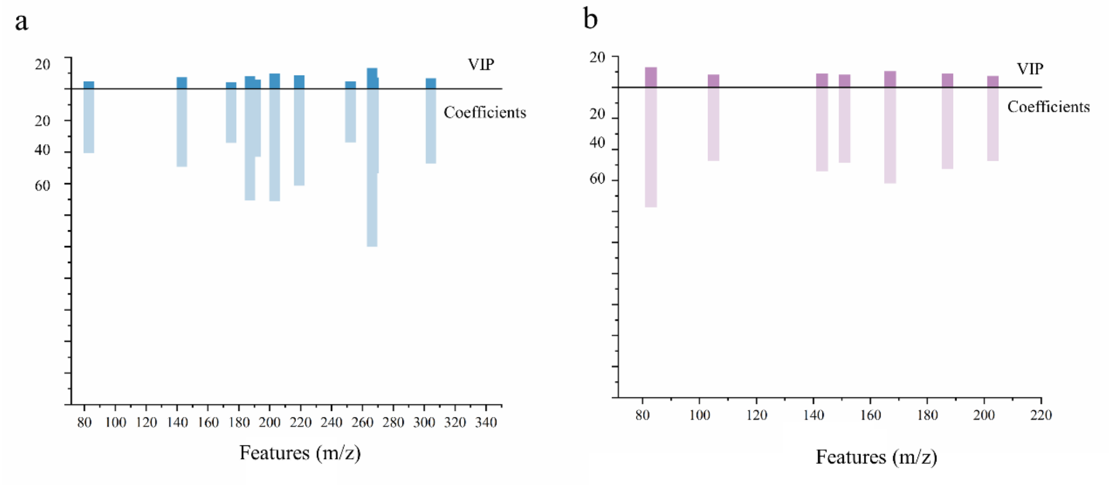

Figure S7. (a) The VIP scores and coefficients distribution of the eleven biomarkers between ARC and CDM. (b) The VIP scores and coefficients distribution of the seven biomarkers between CDM and CHM.

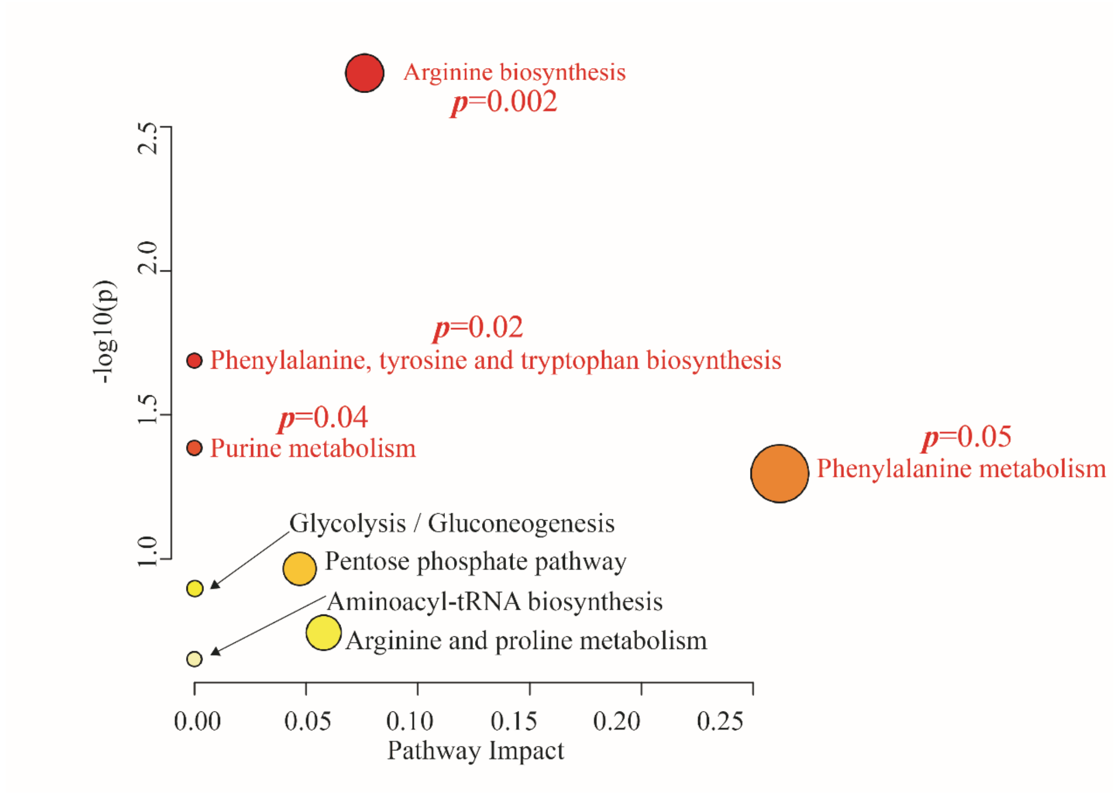

Figure S8. The metabolic pathways of eleven key metabolites in ARC and CDM.

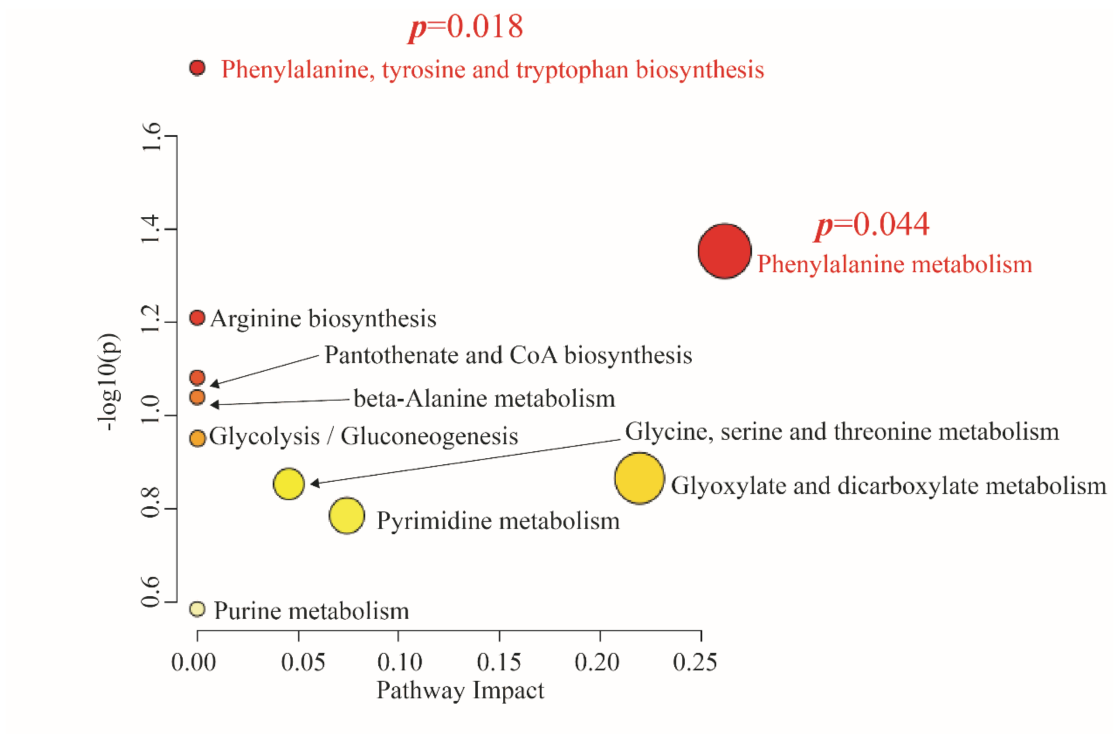

Figure S9. The metabolic pathways of nine key metabolites in ARC and CHM.

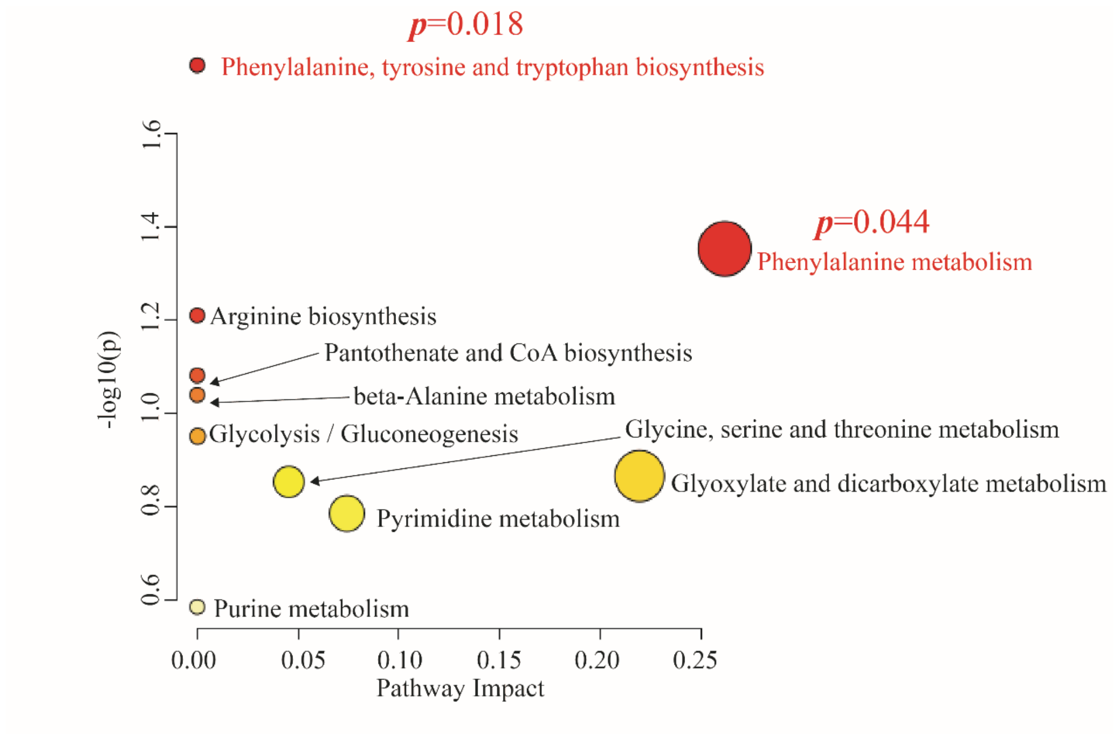

Figure S10. The metabolic pathways of seven key metabolites in CDM and CHM.

Table S1 Sample information of 183 patients including 67 ARC, 51 CDM, and 65 CHM.

AH Sample	Clinical cataract types	Gender Set	Age	Eye axis (mm)	Diabetes
1	ARC	Male	72	<26.5	No
2	ARC	Female	76	<26.5	No
3	ARC	Male	66	<26.5	No
4	ARC	Female	62	<26.5	No
5	ARC	Male	83	<26.5	No
6	ARC	Female	61	<26.5	No
7	ARC	Female	79	<26.5	No
8	ARC	Male	64	<26.5	No
9	ARC	Female	65	<26.5	No
10	ARC	Female	84	<26.5	No
11	ARC	Female	75	<26.5	No
12	ARC	Female	55	<26.5	No
13	ARC	Female	65	<26.5	No
14	ARC	Male	57	<26.5	No
15	ARC	Female	69	<26.5	No
16	ARC	Female	68	<26.5	No
17	ARC	Female	66	<26.5	No
18	ARC	Male	67	<26.5	No
19	ARC	Female	66	<26.5	No
20	ARC	Female	67	<26.5	No
21	ARC	Female	79	<26.5	No
22	ARC	Male	57	<26.5	No
23	ARC	Male	84	<26.5	No
24	ARC	Male	83	<26.5	No
25	ARC	Male	68	<26.5	No
26	ARC	Male	64	<26.5	No
27	ARC	Female	50	<26.5	No
28	ARC	Female	68	<26.5	No
29	ARC	Female	70	<26.5	No
30	ARC	Female	56	<26.5	No
31	ARC	Female	71	<26.5	No
32	ARC	Female	71	<26.5	No
33	ARC	Female	72	<26.5	No
34	ARC	Female	65	<26.5	No
35	ARC	Male	72	<26.5	No
36	ARC	Female	60	<26.5	No
37	ARC	Female	82	<26.5	No

38	ARC	Female	81	<26.5	No
39	ARC	Male	66	<26.5	No
40	ARC	Female	56	<26.5	No
41	ARC	Female	64	<26.5	No
42	ARC	Male	72	<26.5	No
43	ARC	Female	78	<26.5	No
44	ARC	Male	71	<26.5	No
45	ARC	Female	61	<26.5	No
46	ARC	Female	63	<26.5	No
47	ARC	Female	66	<26.5	No
48	ARC	Male	77	<26.5	No
49	ARC	Female	73	<26.5	No
50	ARC	Female	78	<26.5	No
51	ARC	Male	52	<26.5	No
52	ARC	Female	78	<26.5	No
53	ARC	Male	67	<26.5	No
54	ARC	Female	70	<26.5	No
55	ARC	Female	64	<26.5	No
56	ARC	Female	75	<26.5	No
57	ARC	Female	65	<26.5	No
58	ARC	Female	66	<26.5	No
59	ARC	Male	73	<26.5	No
60	ARC	Female	75	<26.5	No
61	ARC	Female	77	<26.5	No
62	ARC	Male	75	<26.5	No
63	ARC	Male	79	<26.5	No
64	ARC	Female	68	<26.5	No
65	ARC	Female	50	<26.5	No
66	ARC	Male	66	<26.5	No
67	ARC	Female	79	<26.5	No
68	CDM	Female	72	<26.5	Yes
69	CDM	Male	56	<26.5	Yes
70	CDM	Male	77	<26.5	Yes
71	CDM	Female	60	<26.5	Yes
72	CDM	Female	68	<26.5	Yes
73	CDM	Male	68	<26.5	Yes
74	CDM	Female	60	<26.5	Yes
75	CDM	Male	59	<26.5	Yes
76	CDM	Female	63	<26.5	Yes
77	CDM	Male	69	<26.5	Yes

78	CDM	Female	71	<26.5	Yes
79	CDM	Female	82	<26.5	Yes
80	CDM	Female	77	<26.5	Yes
81	CDM	Male	64	<26.5	Yes
82	CDM	Male	77	<26.5	Yes
83	CDM	Male	42	<26.5	Yes
84	CDM	Female	55	<26.5	Yes
85	CDM	Male	67	<26.5	Yes
86	CDM	Male	68	<26.5	Yes
87	CDM	Female	73	<26.5	Yes
89	CDM	Female	69	<26.5	Yes
90	CDM	Female	66	<26.5	Yes
91	CDM	Female	73	<26.5	Yes
92	CDM	Female	72	<26.5	Yes
93	CDM	Male	75	<26.5	Yes
94	CDM	Male	63	<26.5	Yes
95	CDM	Female	62	<26.5	Yes
96	CDM	Male	69	<26.5	Yes
97	CDM	Female	55	<26.5	Yes
98	CDM	Female	74	<26.5	Yes
99	CDM	Male	51	<26.5	Yes
100	CDM	Male	81	<26.5	Yes
101	CDM	Female	70	<26.5	Yes
102	CDM	Female	68	<26.5	Yes
103	CDM	Female	66	<26.5	Yes
104	CDM	Male	67	<26.5	Yes
105	CDM	Female	79	<26.5	Yes
106	CDM	Female	67	<26.5	Yes
107	CDM	Male	81	<26.5	Yes
108	CDM	Male	83	<26.5	Yes
109	CDM	Female	82	<26.5	Yes
110	CDM	Female	71	<26.5	Yes
111	CDM	Female	87	<26.5	Yes
112	CDM	Female	71	<26.5	Yes
113	CDM	Female	66	<26.5	Yes
114	CDM	Female	70	<26.5	Yes
115	CDM	Female	73	<26.5	Yes
116	CDM	Male	82	<26.5	Yes
117	CDM	Male	67	<26.5	Yes
118	CDM	Female	73	<26.5	Yes

119	CHM	Male	67	≥26.5	No
120	CHM	Male	51	≥26.5	No
121	CHM	Female	67	≥26.5	No
122	CHM	Male	49	≥26.5	No
123	CHM	Female	62	≥26.5	No
124	CHM	Male	65	≥26.5	No
125	CHM	Male	76	≥26.5	No
126	CHM	Female	65	≥26.5	No
127	CHM	Female	70	≥26.5	No
128	CHM	Female	87	≥26.5	No
129	CHM	Male	65	≥26.5	No
130	CHM	Male	63	≥26.5	No
131	CHM	Male	57	≥26.5	No
132	CHM	Female	56	≥26.5	No
133	CHM	Female	79	≥26.5	No
134	CHM	Male	62	≥26.5	No
135	CHM	Male	62	≥26.5	No
136	CHM	Male	60	≥26.5	No
137	CHM	Male	74	≥26.5	No
138	CHM	Male	61	≥26.5	No
139	CHM	Female	58	≥26.5	No
140	CHM	Female	58	≥26.5	No
141	CHM	Male	51	≥26.5	No
142	CHM	Female	66	≥26.5	No
143	CHM	Female	78	≥26.5	No
144	CHM	Male	57	≥26.5	No
145	CHM	Male	61	≥26.5	No
146	CHM	Male	69	≥26.5	No
147	CHM	Female	66	≥26.5	No
148	CHM	Female	53	≥26.5	No
149	CHM	Female	69	≥26.5	No
150	CHM	Male	73	≥26.5	No
151	CHM	Female	46	≥26.5	No
152	CHM	Male	52	≥26.5	No
153	CHM	Female	67	≥26.5	No
154	CHM	Male	72	≥26.5	No
155	CHM	Male	80	≥26.5	No
156	CHM	Female	49	≥26.5	No
157	CHM	Female	52	≥26.5	No
158	CHM	Female	55	≥26.5	No

159	CHM	Female	71	⩾26.5	No
160	CHM	Female	62	⩾26.5	No
161	CHM	Female	57	⩾26.5	No
162	CHM	Male	65	⩾26.5	No
163	CHM	Male	67	⩾26.5	No
164	CHM	Female	56	⩾26.5	No
165	CHM	Female	65	⩾26.5	No
166	CHM	Female	58	⩾26.5	No
167	CHM	Female	70	⩾26.5	No
168	CHM	Male	57	⩾26.5	No
169	CHM	Male	61	⩾26.5	No
170	CHM	Female	55	⩾26.5	No
171	CHM	Female	69	⩾26.5	No
172	CHM	Female	76	⩾26.5	No
173	CHM	Male	74	⩾26.5	No
174	CHM	Female	69	⩾26.5	No
175	CHM	Male	73	⩾26.5	No
176	CHM	Female	66	⩾26.5	No
177	CHM	Male	74	⩾26.5	No
178	CHM	Female	66	⩾26.5	No
179	CHM	Male	41	⩾26.5	No
180	CHM	Male	61	⩾26.5	No
181	CHM	Female	58	⩾26.5	No
182	CHM	Female	81	⩾26.5	No
183	CHM	Male	84	⩾26.5	No

Table S2 The difference of age/sex among ARC, CDM, and CHM

Characteristics		ARC	CDM	*P* value	ARC	CHM	*P* value	CDM	CHM	*P* value
Age(mean(±SD)		(69.01±8.322)	(69.18±8.758)	0.922[a]	(69.01±8.322)	(64.17±9.511)	0.002[a]	69.18±8.758)	(64.17±9.511)	0.003[a]
Sex	Male	22	21	0.230[b]	22	30	0.083[b]	21	30	0.364[b]
	Female	45	30		45	35		30	35	

[a)] *P* value was calculated by one-way anova; [b)] *P* value was calculated by Chi-square test.

Table S3. The features with $p<0.05$, the top 30 VIP scores and the top 30 coefficient scores. The features marked in bold are matched through HMDB.

ARC and CDM Features (m/z)	ARC and CHM Features (m/z)	CDM and CHM Features (m/z)
80.8516	80.8516	80.8516
**82.9485**	**82.9485**	**82.9485**
98.9487	87.8812	87.8812
**143.035**	90.9206	90.9206
159.019	96.8451	96.8451
**175.037**	103.881	103.881
**187.085**	**104.939**	**104.939**
**190.952**	108.895	108.895
**203.065**	116.962	116.962
203.552	124.895	124.895
**219.066**	**143.035**	**143.035**
221.086	**150.974**	**150.974**
236.201	**166.965**	**166.965**
238.212	**187.085**	**187.085**
**252.206**	**203.065**	**203.065**
**266.21**	**250.219**	360.323
**267.212**	**266.21**	361.344
**304.165**	360.323	376.302
360.323	393.863	381.306
363.34	449.307	393.863
376.302	450.319	449.307
377.316	465.268	450.319
453.142	590.674	465.268
590.674	685.091	590.674
781.734	727.068	685.091
782.741	728.075	781.734
797.696	781.734	781.997
-	782.741	782.741
-	796.007	795.735
-	797.012	796.007

Table S4. The detailed information of the key features.

Query mass	Compound ID	Compound name	Formula	Monoisotopic mass	adduct	Adduct m/z	delta(ppm)
82.9485	HMDB0000294	Urea	CH_4N_2O	60.0323	M+Na	83.0216	880
104.939	HMDB0001352	3-hydroxy-2-oxopropanoic acid	$C_3H_4O_4$	104.0110	M+H	105.0182	754
143.035	HMDB0001366	Purine	C5H4N4	120.0436	M+Na	143.0328	15
150.974	HMDB0000300	Uracil	C4H4N2O2	112.0273	M+K	150.9904	109
166.965	HMDB0041833	Barbituric acid	C4H4N2O3	128.0222	M+K	166.9854	122
175.037	HMDB0000517	L-Arginine	$C_6H_{14}N_4O_2$	174.1117	M+H	175.1190	468
187.085	HMDB0000205	Phenylpyruvic acid	C9H8O3	164.0473	M+Na	187.0366	259
190.952	HMDB00289	Uric acid	$C_5H_4N_4O_3$	168.0283	M+Na	191.0176	343
203.065	HMDB0000122	D-Glucose	C6H12O6	180.0634	M+Na	203.0526	61
219.066	HMDB0000625	Gluconic acid	C6H12O7	196.0583	M+Na	219.0475	84
250.219	HMDB0028780	Cysteinyl-Lysine	C9H19N3O3S	249.1147	M+H	250.1220	388
252.206	HMDB01983	5'-Deoxyadenosine	C10H13N5O3	251.1018	M+H	252.1091	384
266.21	HMDB0029022	Prolyl-Lysine	C11H21N3O3	243.1583	M+Na	266.1475	235
267.212	HMDB0028932	Leucyl-Isoleucine	C12H24N2O3	244.1787	M+Na	267.1679	165
304.165	HMDB0029096	N-Phenylacetylaspartic acid	C16H21N3O3	303.1583	M+H	304.1656	2

[1] K. Lan, Y. Liu, W. Zhang, Y. Liu, A. Elzatahry, R. C. Wang, Y. Y. Xia, D. Al-Dhayan, N. F. Zheng, D. Y. Zhao, *J. Am. Chem. Soc.* **2018**, *140*, 4135.
[2] S. Gibb, K. Strimmer, *Bioinformatics* **2012**, *28*, 2270.
